# You Cannot be Partially Pregnant: A Comparison of Divisible and Nondivisible Outcomes in Delay and Probability Discounting Studies

**DOI:** 10.1007/s40732-015-0144-1

**Published:** 2015-11-03

**Authors:** Przemysław Sawicki, Łukasz Markiewicz

**Affiliations:** Centre for Economic Psychology and Decision Sciences, Economic Psychology Department, Kozminski University, Warsaw, Poland

**Keywords:** Delay discounting, Probability discounting, Money, Medical treatment, Domain specific discounting, Divisibility of alternatives, Divisible and nondivisible medical outcomes, Reverse magnitude effect

## Abstract

Research by *The Psychological Record, 64*(3), 433–440. doi:10.1007/s40732-014-0052-9, (2014) demonstrated the novel finding that the magnitude effect for medical outcomes does not reverse across delay and probability discounting as it does for monetary outcomes. We suggest that a possible reason for the lack of a reverse magnitude effect in nonmonetary outcomes is incomparable divisibility of discounted alternatives.

To test whether the lack of a reverse magnitude effect in probability discounting of medical outcomes is due to incomparable divisibility of treatment effects, 4 studies were conducted. In the replication study, the effect observed by *The Psychological Record, 64*(3), 433–440. doi:10.1007/s40732-014-0052-9, (2014) was marginally not significant, although it was directionally consistent with their prediction of steeper discounting of small medical outcomes (as compared to large, defined as brain cancer) both in time and probability discounting. Our manipulation by substituting a divisible outcome (body paralysis) for an indivisible one (brain cancer) did not, however, bring expected results. We discuss the explanations and possible implications of introduced division for divisible and nondivisible medical outcomes.

Choices in which at least one payment is delayed are referred to as intertemporal choices. In such situations, the decision maker chooses between a smaller earlier or larger later payment. Choices in which at least one payment is only probable are referred to as choices under risk. In such situations, the decision maker considers the choice of certain payment or payment with a given probability (see Madden & Bickel, 2010, for reviews).

A subjective decrease in the value of an amount with an increase of delay or uncertainty is referred to as discounting (Mazur, 1987; Rachlin, Raineri, & Cross, 1991). In the case of intertemporal choices, strong discounting of the delayed amount shows unwillingness to wait and preference for the lower, immediate payment. In the case of choices under risk, strong discounting of the uncertain amount signals aversion to risk and preference for the lower amount without risk.

Existing studies show that the magnitude of an amount affects the process of discounting delayed and risky payments to varying degrees. In the case of intertemporal choices, smaller amounts are discounted more strongly than larger amounts, and in the case of choices under risk, the effect is reversed: larger amounts are discounted more strongly than smaller amounts (Du, Green, & Myerson, 2002; Estle, Green, Myerson, & Holt, 2006; Myerson, Green, Scott, Holt, & Estle, 2003; Vanderveldt, Green, & Myerson, 2015).

The different effects are explained by feelings of disappointment characteristic of choices under risk (Loewenstein & Lerner, 2003). In light of this interpretation, the uncertain larger amount is discounted more strongly than the uncertain smaller amount, since the larger the amount, the greater the potential disappointment when a lottery outcome is unfavorable. In the case of intertemporal choices, both alternatives are certain, so disappointment is not an issue. If there is no risk of disappointment, people prefer to wait and are more willing to wait if the delayed amount is larger.

A recent study of Weatherly and Terrell (2014) showed that reversal of the magnitude effect when discounting probable amounts is a characteristic only of monetary alternatives and that this disappears when discounting nonmonetary alternatives. In a series of studies the researchers focused on delay and probability discounting of monetary ($100 or $100,000) and medical (acne or brain cancer) outcomes. Although for monetary outcomes they replicated results observed in the literature (the magnitude effect – steeper discounting of the small outcome than the large outcome in the case of delay discounting; and the reverse magnitude effect – steeper discounting of the large outcome than the small outcome for probability discounting), they did not find a reverse magnitude effect for probability discounting in the case of medical outcomes. The above results raise the question of whether discounting of nonmonetary alternatives is subject to different discounting processes than discounting of monetary alternatives or whether Weatherly and Terrell’s (2014) results were an artefact of the procedure used.

We argue that a possible reason for the lack of a reverse magnitude effect in nonmonetary outcomes is incomparable divisibility of treatment effects in Weatherly and Terrell’s approach. There are plenty of similar situations in real life: a person would probably accept a different level of risk in a competition if all players were compensated according to their performance (the result therefore being divisible) compared to a situation where one winner takes everything (a nondivisible result). Similarly, having one ski probably has as much utility as having no ski at all, since, when compared to having two skis, having one ski is almost useless, and its utility is close to zero. This can be understood in terms of a dramatically different curvature in the utility function for different goods. Having half of an initial monetary value gives you more or less half of the initial utility (depending on your money-utility function), while there are some goods where utility is either all or nothing. A similar type of reasoning is probably also applicable in many domains not involving money or goods (e.g., with respect to ailments such as brain cancer in the medical domain).

Weatherly and Terrell (2014) used acne and brain cancer as discounted medical states. However, in our opinion, these states differ not only in magnitude (of potential negative effects) but also in divisibility. High divisibility applies in the case of acne. Treatment efficacy translates into the number of pimples on the face. In this sense, acne treatment effects are divisible (and having half as many pimples probably makes one feel better). But the situation is different in the case of brain cancer: here we cannot speak about divisibility of treatment effects. As in the case of pregnancy, one cannot have brain cancer partially. If the treatment does not treat the cancer fully, for the person being treated the risk of death still exists (thus, having a brain tumor half as small as it was initially does not make one much happier). If the risk remains, the person involved is motivated to overvalue treatment efficacy, which influences the discounting rate so that increased treatment efficacy translates into a decrease of the discounting rate.

Thus, we suggest that the lack of divisibility of treatment effects in the case of brain cancer resulted in Weatherly and Terrell’s failure to obtain the reverse magnitude effect for nonmonetary outcomes. We hypothesized that replacement of brain cancer with a disease whose treatment effects are divisible (e.g., body paralysis) would restore the reverse magnitude effect for nonmonetary outcomes (having full body paralysis is probably far worse than having one’s legs paralyzed, which is far worse than having one leg paralyzed). We therefore aimed to replicate Weatherly and Terrell’s (2014) finding using a nongradable disease, and hypothesized that:

- **H1.** In the case of a nongradable disease (brain cancer), the magnitude effect for medical outcomes does not reverse between delay and probability discounting as it does for monetary outcomes.

We also aimed to demonstrate that the effect disappears in the case of a gradable disease (e.g., partial body paralysis) and hypothesized that:

- **H2:** In the case of a gradable disease (partial body paralysis), the magnitude effect for medical outcomes reverses between delay and probability discounting, as it does for monetary outcomes.

## Method

### Research Design

The study protocol was approved by the Institutional Review Board at Kozminski University. To test H1 and H2, four studies were conducted (see Table 1). The first two concerned delay discounting and the last two concerned probability discounting. Studies 1 and 3 were exact replications of Weatherly and Terrell’s (2014) experiments. In Studies 2 and 4, brain cancer was replaced by body paralysis.

**Table 1:** Summary of conducted studies

	DELAY DISCOUNTING		PROBABILITY DISCOUNTING	
	Money	Medical	Money	Medical
Study 1	$100, $100’000	Acne, Brain cancer		
Study 2	$100, $100’000	Acne, Body paralysis		
Study 3			$100, $100,000	Acne, Brain cancer
Study 4			$100, $100,000	Acne, Body paralysis

All four studies were conducted in parallel using the Amazon Mechanical Turk (MTurk) and the LimeSurvey platform. Only people with at least a 95 % prior approval rate were invited to participate. Shortly after giving their informed consent, participants were randomly distributed to one of the four studies mentioned in Table 1. The average time taken to perform the study was *M* = 9.39 minutes (*SD* = 4.41; with no significant differences between experimental conditions). All participants received a payment of $0.40 USD.

### Procedure for Studies 1 and 2: Delay Discounting Investigations

Study 1 was an exact replication of the experiments done by Weatherly and Terrell (2014). In Study 2, brain cancer was replaced by body paralysis.

When medical treatments were discounted participants read the following instructions:

*Suppose you were suffering from acne (brain cancer/body paralysis). Your physician informs you that you will need to wait X (time) before obtaining a treatment that is 100 % effective. However, you could immediately begin a treatment that has a lesser chance of success. What minimum percent chance of success would you be willing to accept for the different treatment in order to choose it rather than waiting X (time)?*

When monetary outcomes were discounted, participants read the same in both studies:

*If you won $100 ($100,000) and were not going to get the money for X (time), what is the smallest amount of money you would accept today rather than having to wait X (time)?*

Similarly to the original study, the question was repeated 5 times in succession to gather answers for 5 delay periods (6 months, 1 year, 3 years, 5 years, 10 years – the periods being presented in random order), for each outcome size (large vs. small magnitude), for each domain (monetary vs. medical). Thus, in total 20 questions were asked at this stage (5 × 2 × 2). The order of particular delay periods was randomized within each of four blocks (medical small, medical large, monetary small, monetary large); also, the blocks themselves were presented in random order. Participants were able to move to another block only after answering all five questions from a previous block; however, at any stage participants were allowed to use a “back” button to inspect and correct previous answers. We used a slider-type scale, with end points labeled 0 and 100 % for the medical conditions and 0 to $100 / $100,000 for the monetary conditions; 2 % increments (e.g., 2 %, $2, or $2,000) were used to gather answers instead of the radio button cafeteria-type scale used in the original study. In an online approach, including 50 radio buttons within one column would require scrolling to access higher values (the possibility of anchoring therefore existing), while breaking things into two columns could have resulted in anchoring answers with respect to the top of each column.

### Procedure for Studies 3 and 4: Probability Discounting Investigations

Study 3 was an exact replication of the experiments performed by Weatherly and Terrell (2014). In Study 4, brain cancer was replaced by body paralysis.

When monetary outcomes were discounted, participants read the following instructions:

*You are a finalist in a national sweepstake. You have a X% chance of winning $100 ($100,000). However, if your number is not called, you do not receive anything. The organization running the sweepstake is willing to guarantee to pay you a certain amount of money if you would agree to remove your name from the sweepstake. What is the smallest amount of money you would be willing to accept rather than having a X% chance of winning $100 ($100,000)?*

When medical treatments were discounted, participants read the same in both studies:

*Suppose you were suffering from acne (brain cancer). Your physician informs you that there are two treatment options. The first one completely cures the disease, but it only works for X% of the patients who choose it. The second treatment is guaranteed to work, but it only partially treats the disease. You can only afford to choose one treatment. What is the minimum percentage of success that the second treatment would need to guarantee for you to choose it over the first treatment?*

Again, we used a slider-type scale, with endings labeled 0 and 100 % for the medical conditions and 0 to $100 / $100,000 for the monetary conditions; 2 % increments were used (e.g., 2 %, $2, or $2,000). Similar to the original study, each question was repeated 5 times in a row to gather answers for five probability levels – 1 %, 10 %, 50 %, 90 %, and 99 % – all presented in random order, for each outcome size (large vs. small magnitude), for each domain (monetary vs. medical). Thus, again, in total 20 questions were asked at this stage (5 × 2 × 2). The order of particular probability levels was randomized within each of the four blocks (medical small, medical large, monetary small, monetary large), and the blocks themselves were also presented in random order. Again, participants were able to move to the next block only after answering all five questions from a previous block; however, at any stage participants were allowed to use a “back” button to inspect and correct previous answers.

### Data Analysis

Exclusionary criteria were identical to those used by Weatherly and Terrell (2014). We identified instances in which indifference points were not monotonically decreasing with time or probability (e.g., participants were excluded when they wanted to receive more for longer than for shorter delays, or wanted to receive more for less probable than more probable results). Thus, with respect to all outcomes, for any single participant, no indifference point could be greater than the one immediately preceding it.

Data for participants whose choices met the criterion for systematic discounting were analyzed by calculating the area under the curve (AUC; Myerson, Green, & Warusawitharana, 2001) using the formula (a_2_ – a_1_)[(b_1_ + b_2_)/2], where standardized a_1_ and a_2_ are consecutive delays in months (or probabilities) while b_1_ and b_2_ represent consecutive subjective values of delayed (or uncertain) payoffs. This method allows calculation of the speed of a value’s decrease as the delay (or uncertainty) increases. The lower the AUC, the greater the diminution from the starting value because of delaying (or making more uncertain) the starting value.

### Closing Questionnaire

To show that the results presented by Weatherly and Terrell (2014) depended on the divisibility of the medical condition we conducted a manipulation check at the end of each study. Participants were asked about their hypothetical worry (using a slider-type question with responses on a 0- to 100-point scale) if suffering from both low- and high-intensity variants of acne, brain cancer, or body paralysis. Moreover, participants were asked to provide demographic information and asked whether they had any history of medical conditions related to those researched in the study. Additionally, to validate the Amazon Turk sample, at the end of the questionnaire, a question applying a new tool for detecting participants who had not followed instructions was added: the instructional manipulation check (IMC), as suggested by Oppenheimer, Meyvis, and Davidenko (2009). This asked participants about sports activities they engaged in regularly, with lengthy and boring instructions explaining why it was so important, and at the end there was a request in small print for participants to ignore the question and type their year of birth into a text box below.

## Results

### Studies 1 and 2: Delay Discounting Investigations

The original sample of participants consisted of 167 people enrolled from Amazon’s MTurk worker pool.^1^ After employing the exclusionary criteria (which were exactly the same as in Weatherly and Terrell’s, 2014, study) the final sample of participants for Studies 1 and 2 consisted of 86 people (see Table 2).

**Table 2:** Details of participants in Studies 1 and 2 before and after employing exclusionary criteria

	Original sample				After employing exclusionary criteria				
	*N*	Males %	Age *M*	Age *SD*	*N*	Males %	Age *M*	Age *SD*	Reduction
Study 1	85	62	35.17	11.15	42	60	37.43	12.27	50.59 %
Study 2	82	54	35.83	11.02	44	59	35.93	10.99	46.34 %

The exclusion rates for the delay discounting studies (Studies 1 and 2) reported in Table 2 were similar to the 49 % rate reported in the study of Weatherly and Terrell (2014).^2^

**Fig. 1:**
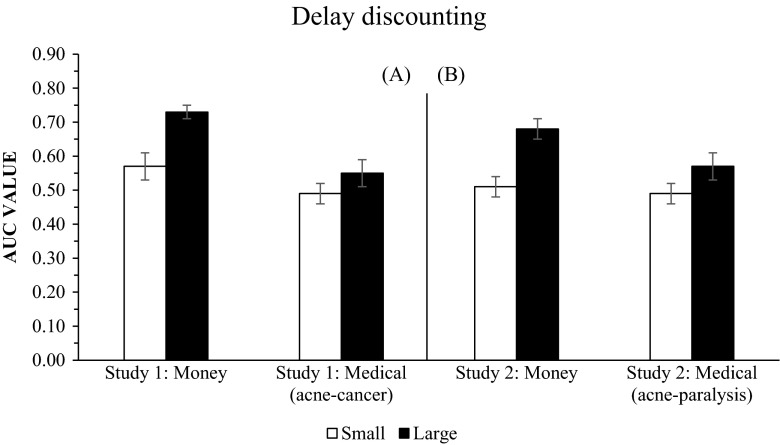
Mean AUC values for each outcome discounted in Study 1 (on the left, panel **a**) and Study 2 (on the right, panel **b**). The error bars represent one standard error of the mean for all participants

Figure 1 presents the mean AUC values for the small and large magnitude monetary and medical-treatment outcomes in the delay discounting conditions. The left panel, A, presents the results of Study 1 (a pure replication of Weatherly and Terrell’s study, with brain cancer as the “large magnitude” medical condition), while Panel B, on the right, presents the Study 2 results, with brain cancer replaced by body paralysis.

In all groups, the larger outcome was discounted more steeply than the smaller outcome. In Study 1, a repeated measures ANOVA on rates of discounting identified a statistically significant difference in discounting strength between the smaller and larger monetary amounts, *F*(1, 41) = 31.11; *p* = .001; η^2^ = .431. However, the difference in discounting strength between the smaller and larger magnitude medical outcomes (acne vs. brain cancer) was marginally nonsignificant, *F*(1, 41) = 2.94; *p* = .094; η^2^ = .067. In Study 2 we found a higher discount rate for smaller monetary outcomes than larger outcomes, *F*(1, 43) = 24.09; *p* = .001; η^2^ = .359, as well as for the smaller as compared to the larger magnitude medical condition, *F*(1, 43) = 7.41; *p* = .009; η^2^ = .147.

Moreover, the results showed statistically significance differences between the large monetary and large magnitude medical outcomes in Study 1: $100,000 vs. brain cancer, *F*(1, 41) = 24.53; *p* = .001; η^2^ = .374, and in Study 2: $100,000 vs. body paralysis, *F*(1, 43) = 5.84; *p* = .020; η^2^ = .120. This suggests that large medical conditions are discounted more strongly than large monetary outcomes. However, this was not the case for small outcomes: In both Study 1, *F*(1, 41) = 3.50; *p* = .068; η^2^ = .079, and Study 2, *F*(1, 43) = .24; *p* = .627; η^2^ = .006, differences in discounting strength for small magnitude medical outcomes (acne) and small monetary outcomes ($100) were nonsignificant (*p* > .05).

### Studies 3 and 4: Probability Discounting Investigations

The original sample of participants consisted of 143 people enrolled from Amazon’s MTurk worker pool. After employing the exclusionary criteria, the final sample across both studies consisted of 97 participants (see Table 3).

**Table 3:** Details of participants in Studies 3 and 4 before and after employing exclusionary criteria

	Original sample				After employing exclusionary criteria				
	*N*	Males %	Age *M*	Age *SD*	*N*	Males %	Age *M*	Age *SD*	Reduction
Study 3	75	55	34.12	11.25	50	54	35.94	10.92	33.33 %
Study 4	68	53	32.49	12.11	47	53	32.74	13.18	30.88 %

The exclusion rates for the probability discounting studies (Studies 3 and 4) reported in Table 3 were identical to that reported in Weatherly and Terrell’s (2014) study (28 %).^3^

**Fig. 2:**
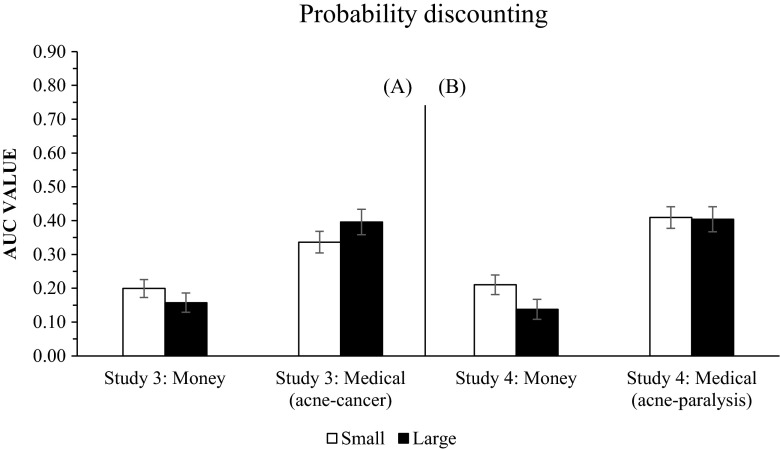
Mean AUC values for each outcome discounted in Study 3 (left, Panel **a**) and Study 4 (right, Panel **b**). The error bars represent one standard error of the mean for all participants

Figure 2 presents the mean AUC values for the small and large magnitude monetary and medical-treatment outcomes in the probability discounting conditions: Panel A, on the left, depicting the Study 3 results (the replication study), and Panel B, on the right, depicting the Study 4 results (with the brain cancer condition replaced by the body paralysis condition).

In Study 3, participants more strongly discounted larger monetary outcomes when compared to smaller monetary outcomes, *F*(1, 49) = 8.09; *p* = .006; η^2^ = .142. Although from Figure 2 it might appear that smaller magnitude medical outcomes (acne) were discounted more steeply than large medical outcomes (brain cancer), the repeated measures ANOVA showed that this was not the case – the difference was marginally not significant, *F*(1, 49) = 3.38; *p* = .072; η^2^ = .064. In Study 4, participants more strongly discounted larger monetary outcomes when compared to smaller monetary outcomes, *F*(1, 46) = 19.65; *p* = .001; η^2^ = .299. For medical outcomes (body paralysis vs. acne), however, the difference in discounting strength was not significant, *F*(1, 46) = .04; *p* = .847; η^2^ = .001.^4^

Results also showed statistically significant differences between large magnitude monetary and large magnitude medical treatment outcomes in both Study 3 ($100,000 vs. brain cancer), *F*(1, 49) = 33.95; *p* = .001; η^2^ = .409, and Study 4 ($100,000 vs. body paralysis), *F*(1, 46) = 39.98; *p* = .001; η^2^ = .465. Finally, significant differences were also found between small magnitude medical treatment outcomes and small monetary outcomes in Study 3 ($100 vs. acne), *F*(1, 49) = 12.548; *p* = .001; η^2^ = .204, and Study 4 ($100 vs. acne), *F*(1, 46) = 34.113; *p* = .001; η^2^ = .426. These results all suggest that monetary outcomes are discounted more strongly than medical outcomes when comparing small–small and large–large magnitude outcomes.

### Manipulation Check

After the 20 discounting-related questions, participants were presented with manipulation check questions involving the rating of worry (on a 0 to 100 scale) for items mentioning both small intensity illness (e.g., “Imagine that a doctor diagnosed you with body paralysis of low intensity”) and large intensity illness (e.g., “Imagine that a doctor diagnosed you with body paralysis”). Thus, participants had to rate 3 medical conditions × 2 intensity states, making a total of six questions presented in random order to participants. We assumed that the reduction of worry (between high and low intensity) should be higher for a divisible illness as compared to an indivisible one. The results confirmed our expectations (see Table 4).

**Table 4:** Manipulation check: Hypothetical worry when suffering from acne, brain cancer, or body paralysis (of both low and high intensity). Base: All respondents participating in Studies 1 to 4, *N* = 183

	LOW intensity		HIGH intensity		Difference			
	*M*	*SD*	*M*	*SD*	*M*	*SD*	*T*	*p*
Acne	18.28	19.50	42.24	28.48	-23.96	22.34	-14.50	<.001
Brain cancer	87.35	19.32	96.60	12.39	-9.25	17.11	-7.31	<.001
Body paralysis	77.05	24.56	93.41	13.86	-16.36	19.99	-11.07	<.001

Indeed, participants rated high-intensity cancer higher on the worry scale than low-intensity cancer (a difference of 9.25 points on the 0–100-point scale); they also did the same for body paralysis (by 16.36 points) and for acne (by 23.96 points, on average). However, the decrease in worry between the high and low conditions was the highest for the easily graduated condition of acne, smaller for body paralysis, and lowest for brain cancer (as demonstrated by paired *t* tests: *p* < .001 in all cases). Thus, we conclude that in general receiving treatment that partially treats brain cancer results in less happiness than receiving treatment that partially treats (by the same proportion) body paralysis or acne.

Moreover, our study sample was highly disciplined with respect to reading instructions. Only 7 out of 183 participants failed to read long and boring instructions carefully, providing an answer to the first part of the question rather than acceding to the final request in the concluding IMC task (Oppenheimer et al. 2009): this can be interpreted as a successful sample validation.

## General Discussion

In the novel titled *The Gambler,* published by Fyodor Dostoyevsky (1996) in 1866, the main character takes high risks in a casino and does not leave the roulette table with money that is already won because he needs a specific sum of money to pay the debts of a lady he is in love with. He is not interested in any sum smaller than this amount because a lower amount will not help. In short, there are situations where only a complete outcome, and not a partial outcome, matters. Such situations do not only occur in fiction.

Weatherly and Terrell (2014) argued that in time discounting both medical and monetary outcomes, small outcomes are discounted more steeply than large outcomes. It should be noted, however, that the repeated measures ANOVA reported for time discounting on p. 436 of their paper, “*F*(1, 81) = 3.95, *p* = 0.050, η^2^ = 0.046”, had a very small effect size associated with it and that *p* was close to (if not equal to) a nonsignificant value. We replicated Weatherly and Terrell’s study and demonstrated that, for money discounting, small amounts are discounted more steeply than larger amounts, but that a similar phenomenon does not occur for medical outcomes when large and small are defined as cancer and acne, respectively, *F*(1, 41) = 2.94; *p* = .094; η^2^ = .067. However, when using divisible medical outcomes (paralysis and acne), so that the situation vis-a-vis divisibility was comparable to that for money, we found an effect similar to that obtained for monetary outcomes, with a higher discount rate for smaller than for larger medical outcomes, *F*(1, 43) = 7.41; *p* = .009; η^2^ = .147. This confirmed our expectation that divisible medical outcomes would behave in the same way as divisible monetary outcomes.

For probability discounting, Weatherly and Terrell observed differences in the discounting of monetary and medical outcomes. Large monetary values were discounted more steeply than small values, but small medical outcomes were discounted more steeply than large outcomes. However, the effect size associated with the repeated measures ANOVA for medical outcomes (where large was defined as cancer and small as acne) on p. 438 of Weatherly and Terrell’s paper, “*F*(1, 129) = 4.94, *p* = 0.028, η^2^ = .037,” again indicated a very small observed effect, and the effect was not observed at all in our replication study, F(1, 49) = 3.38; *p* = .072; η^2^ = .064. After substituting nondivisible cancer with divisible paralysis, the effect was negligible, *F*(1, 46) = .04; *p* = .847; η^2^ = .001, with large medical outcomes discounted at the same rate as small medical outcomes. Hence, the manipulation introduced did not alter probability discounting in the manner expected.

The manipulation-check part of the study showed that the (dis)utility drop associated with declining illness levels (from high to low intensity) is smaller for some severe illnesses (such as cancer) than for other severe illnesses (such as paralysis), with the former being less divisible than the latter. Therefore, people would probably behave differently when (as in Studies 3 and 4) choosing between two treatments, one of which completely cures the disease but works for only a certain percentage of the patients who choose it, and one of which is guaranteed to work but only partially treats the disease (as expressed by a certain percentage). Having a small brain tumor is almost equally as bad as having a large one: a person can die from either. Therefore, partial tumor reduction would probably be avoided in favor of even a small chance of full recovery, and a disproportionately greater percentage chance of success would be demanded in the case of the latter treatment to compensate for even a small chance of full recovery offered by the former treatment. Ultimately, in this situation, a patient should be less impulsive compared to a patient suffering from a divisible illness such as paralysis. Thus, although Weatherly and Terrell’s (2014) testing of their hypothesis was problematic, and although we did not manage to replicate their findings, the rationale for the hypothesis tested still remains appealing: for nondivisible medical outcomes people might engage in steeper small discounting rather than large probability discounting, but for divisible medical outcomes they should behave as they do for divisible monetary outcomes – with steeper discounting of large payoffs than small payoffs.

We believe that a particularly complicated answering paradigm could at least partially explain such results. Respondents usually have trouble when scaling their attitudes on ratio scales: when asked open ended questions such as “What is the smallest amount of money (minimum percentage of success) you would be willing to accept,” they could treat the whole situation as a bargaining process and make high declarations because more money (or a greater chance of success) is always better than less. In consequence, scale sensitivity deteriorates significantly. Thus, in further replication studies other choice based methods should be used, such as (1) adjusting procedures (often referred to as multiple-choice methods; Weatherly, 2014; Weatherly & Derenne, 2011, 2013) or (2) the choice-based conjoint method (Bialek, Markiewicz, & Sawicki, 2015; Czupryna, Kubińska, & Markiewicz, 2014).

Differences in results between our study and Weatherly and Terrell’s study can be partially attributed to sample characteristics. While the two experiments in the latter study recruited from a University participant pool within a college population (mean ages 20 and 20.8 years; 68 % and 84 % females), our sample consisted of a (more diverse) group of AMT volunteers. Moreover, our sample was older and better balanced in terms of gender (*M* = 36.46 years, *SD* = 11.90; 44 % females). Because of the age difference, our participants may have been more sensitive to medical outcomes (the relevance of this topic to participants would be likely to increase with age) or generally more patient. Also, it has been suggested that in general AMT workers are more rational than samples drawn from other populations (Hardisty, Thompson, Krantz, & Weber 2013b). In fact, the present impressive results for the IMC task show that our sample carefully read the instructions, paying attention to details, and support the idea that the AMT is a valuable tool for conducting discounting studies (Goodman, Cryder, & Cheema, 2013).

### Limitations

In the interests of fully replicating Weatherly and Terrell’s (2014) study we followed their approach while consciously acknowledging its limitations. Thus, similarly to Weatherly and Terrell, we used a simplified version of Johnson and Bickel’s (2008) exclusion criteria, although it might be argued that all exclusion criteria should have been applied. We also used the same delay periods as Weatherly and Terrell (6 months, 1 year, 3 years, 5 years, and 10 years). A potential limitation here is that a person with brain cancer would probably not want to wait for treatment and that a large amount of discounting would occur between 1 day and 6 months. Equivalents of monetary outcomes were provided on a different scale (in money) than equivalents for medical outcomes (in percentages). Thus, making direct comparisons is questionable. We did not measure approximate money equivalents for a given disease – it might questioned whether they are comparable even though there are studies controlling this parameter (e.g., Chapman, 1996). Also, the scenario provided to participants, by virtue of its complicated choice structure, may have been difficult for participants to understand (resulting in quite high exclusion rates). Finally, participants may have been incapable of imagining that they were suffering from brain cancer or paralysis.

## Footnotes

1 The MTurk sample was also used successfully for previous discounting studies (Hardisty, Appelt, & Weber 2013a)

2 Using the algorithm for identifying nonsystematic data proposed by Johnson and Bickel (2008) resulted in exclusion rates of 53 % and 49 % for Studies 1 and 2, respectively. However, in the interests of comparability, we used the simplified criterion proposed by Weatherly and Terrell (2014).

3 Using the algorithm for identifying nonsystematic data proposed by Johnson and Bickel (2008) resulted in exclusion rates of 32 % and 25 % for Studies 3 and 4, respectively, but, again, in the interests of comparability, we used the simplified criterion proposed by Weatherly and Terrell (2014).

4 Since transforming probability into odds against is known to heavily influence AUC by responses at the longest odds, we recalculated AUC data with no transformation employed. The results of Experiments 3 and 4 would not have differed had we employed this method of calculating AUC.
